# Novel Genetic Associations and Range of Phenotypes in Children with Disorders of Sex Development and Neurodevelopment: Insights from the Deciphering Developmental Disorders Study

**DOI:** 10.1159/000447958

**Published:** 2016-09-02

**Authors:** Gabriella Gazdagh, Edward S. Tobias, S. Faisal Ahmed, Ruth McGowan, DDD Study Group

**Affiliations:** ^a^West of Scotland Regional Genetics Service, Laboratory Medicine Building, Queen Elizabeth University Hospital, Glasgow, UK; ^b^Academic Unit of Medical Genetics and Clinical Pathology, College of Medical Veterinary and Life Sciences, University of Glasgow, Glasgow, UK; ^c^Developmental Endocrinology Research Group, Royal Hospital For Children, University of Glasgow, Glasgow, UK; ^d^Wellcome Trust Sanger Institute, Wellcome Trust Genome Campus, Hinxton, Cambridge, UK

**Keywords:** DDD study, Disorders of sex development genes, Genetic associations, Mutations

## Abstract

A range of phenotypes that are associated with disorders of sex development (DSD) may also be encountered in patients with neurodevelopmental delay. In this study we have undertaken a collaborative retrospective review of anonymised phenotypic and genotypic data from the UK-wide Deciphering Developmental Disorders (DDD) study. Our objectives were to determine the frequency and range of DSD phenotypes observed in participants in the DDD study and to identify novel genetic associations. We found that of 7,439 DDD participants, 603 (8%) had at least one genital abnormality. In addition, we found that DSD occurs in 5% of patients with learning difficulties. Causative mutations were found in 13 developmental genes, of which, crucially, 6 had no previous reported association with DSD. Our findings indicate that recognition of these associations should not be overlooked in the management of patients with complex conditions and that exomic sequencing through projects like DDD increases diagnostic yield.

Disorders of sex development (DSD) are a group of conditions affecting the reproductive system that commonly present in early infancy and may arise as a result of gonadal, adrenal, or hormonal dysfunction. The overall birth prevalence has been reported to be as high as 1:300, but truly atypical genitalia are less common, estimated at 1:3,000 [Ahmed et al., 2011]. It is recognized that DSD can be isolated or may be associated with a variety of other conditions [Cox et al., 2014]. A recent survey of 650 affected cases in the I-DSD Registry (www.i-dsd.org) has shown that ∼27% of cases are associated with an additional congenital abnormality [Cox et al., 2014]. Our understanding about the underlying genetic basis of DSD conditions is limited. Regions of genomic imbalance and mutations in several genes have been found to be associated with DSD, but in at least half of the cases no molecular diagnosis is made. Detection of a molecular cause is important as it can assist in syndrome detection, in determining precise diagnosis, and in ascertaining likely prognosis with regard to potential infertility and tumour risks [Kyriakou et al., 2015]. Furthermore, it can inform crucial management decisions regarding, for example, sex of rearing and endocrine therapy.

Since April 2011, the UK-wide Deciphering Developmental Disorders (DDD) study has collected DNA and clinical information from over 13,000 undiagnosed children and adults presenting with a developmental disorder to 24 UK regional genetics services. The primary aim of that study has been to identify the underlying molecular diagnosis by performing detailed genetic testing using SNP array-CGH, and exome sequencing on the samples obtained from the family trios [Wright et al., 2015]. The DDD study has also enabled the creation of a carefully curated list of genes reported to be associated with developmental disorders, the DDG2P (Developmental Disorders Genotype-to-Phenotype) database [Deciphering Developmental Disorders Study, 2015].

In this study, the objectives were, firstly, to determine the prevalence of DSD within the current DDD cohort and the range of DSD phenotypes in children with undiagnosed neurodevelopmental disorders and, secondly, to identify the genetic and syndromic associations with DSD.

## Material and Methods

### Phenotypic Data Collection

The phenotypic data collated by the DDD study were entered by clinical geneticists who recruited the patients into the study. These features were entered through the DDD study's secure website, Decipher. Human Phenotype Ontology (HPO) terms were used to standardize the description of the observed features of the patients; hence, the choice of feature description was limited to the terms available within HPO. Phenotypic data from children and adults recruited from April 2011 until May 2014 with at least one HPO term under ‘Abnormality of the genital system’ were analysed. All cases that were reported as hypoplastic male genitalia or genital hypoplasia are described as genital hypoplasia in this report.

### Genetic Data Acquisition

From the first 1,133 family trios to have their exome sequence data analysed, the Variant Call Format (VCF) files from 81 family trios with ‘Abnormality of the genital system’ and the details of the pathogenic genetic variants reported back to the clinicians were reviewed, utilising strategies that have been described previously [Gilissen et al., 2012].

The gene mutations analysed were those which had been detected by the analysis, by exomic DNA sequencing, of trios consisting of affected offspring and their unaffected parents for de novo mutations predicted to be functionally significant, as described previously [Wright et al., 2015]. Essentially, sequencing was performed using Agilent SureSelect 55MB Exome Plus with Illumina HiSeq technology, identification of de novo variants by the DeNovoGear program, and the annotation of the single nucleotide variants (SNVs) by the variant effect predictor (VEP) software of the Ensembl database (http://www.ensembl.org/Tools/VEP). Subsequent validation of de novo SNVs was undertaken by targeted Sanger DNA sequencing [Wright et al., 2015].

## Results

**Fig. 1:**
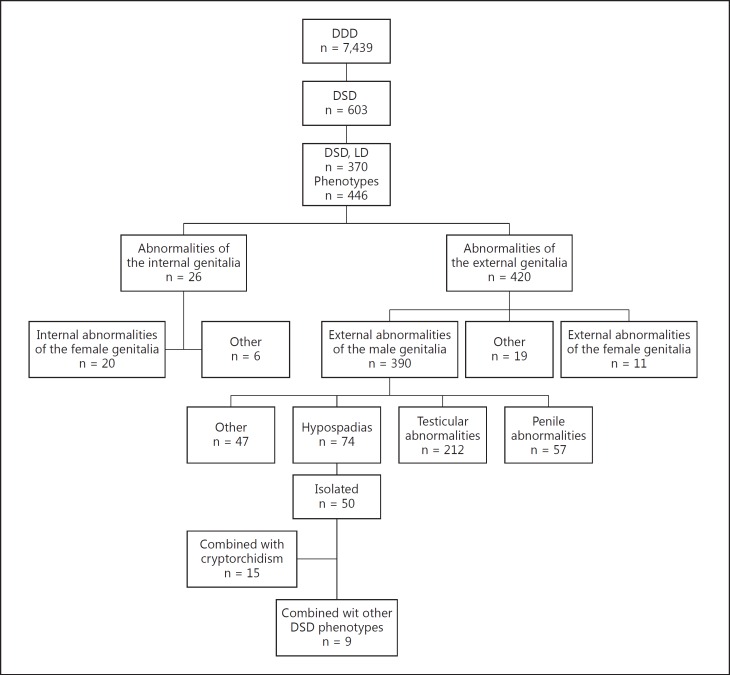
Patients recruited to the DDD DSD study, phenotypic data, from top down: DDD patients (n = 7,439), DSD phenotypes (n = 603), DSD + LD (n = 370). Further distributions of DSD phenotypes are described.

A total of 603 (8%) of the 7,439 DDD study participants recruited to DDD at the time of analysis were found to have at least one HPO term under ‘Abnormality of the genital system’. The most common associated feature in these patients with DSD was Learning Difficulties (LD). We selected the individuals with LD (n = 370) to review the range of their DSD phenotypes (fig. 1).

### Phenotypes

Of the 603 children with DSD, 370 (61%) had at least one ‘neurodevelopmental delay’ diagnosis. Across DDD, around 87% (6,472 of 7,439 DDD participants) have intellectual disability or developmental delay [Wright et al., 2015]. Therefore, in this study, the proportion of individuals with learning disability who were also found to have DSD was 370 out of 6,472 (i.e., ∼5%).

**Fig. 2:**
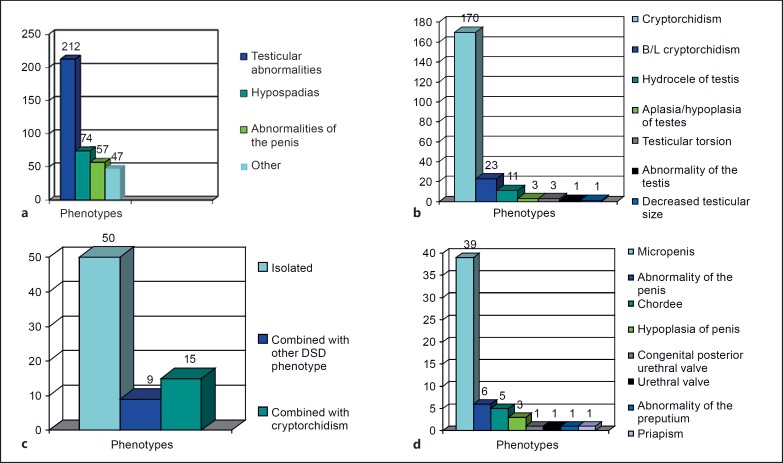
Phenotypic characteristics found in the patient cohort. **a** Abnormalities of male external genitalia. **b** Testicular abnormalities. **c** Hypospadias. **d** Penile abnormalities.

In the group of 370 patients, there were a total of 446 DSD phenotypes reported, of which the majority, 420 (94%), were abnormalities of the external genitalia. Of the 420 external genitalia abnormalities, 390 were male, 11 female abnormalities, and 19 other phenotypes. More specifically, of the male external genitalia abnormalities, 212 (54%) were testicular, 74 (19%) were hypospadias, 57 (15%) were penile, and 47 (12%) were other abnormalities (fig. 2a). The majority of the ‘other’ phenotypes included scrotal abnormalities. Within the ‘testicular abnormalities’ group of phenotypes, cryptorchidism, bilateral cryptorchidism, hydrocele, and other phenotypes were observed (fig. 2b). Bilateral cryptorchidism was described in 23 subjects, whereas in most of the patients with cryptorchidism (170), laterality was undefined. The majority of cases of hypospadias occurred as an isolated finding (67.5%), however, 15 of the 74 (20%) cases occurred together with cryptorchidism and 9 cases were present in combination with other DSD phenotypes (fig. 2c). Micropenis was the most common penile abnormality found in these patients (fig. 2d).

### Genotypes

Pathogenic mutations were found in 14 genes already listed in the Developmental Disorders Genotype Phenotype (DDG2P) database (https://decipher.sanger.ac.uk/), consistent with a range of syndromic diagnoses previously reported to have associated DSD features including KBG syndrome [Tekin et al., 2004], Meier-Gorlin syndrome ([de Munnik et al., 2012], alpha-thalassemia/mental retardation syndrome, Kabuki syndrome, and Donnai-Barrow syndrome (table 1). We have excluded one of these patients from our study as subsequent to phenotype clarification with the clinician he was found to have solely penoscrotal web. Of these likely pathogenic mutations, however, 6 of 13 (46%) were found in DDG2P genes not previously reported to be associated with DSD (table 2).

## Discussion

DSD occur in a proportion of children with neurodevelopmental delay, but the range of these DSD phenotypes has not previously been described. Exploring these syndromic and genetic associations will increase our knowledge about the pathogenesis of DSD and enable more accurate genetic counselling for the families. In addition, the determination of precise genetic abnormalities underlying the phenotypes can facilitate an improved prediction of prognosis and the ability to make appropriate management decisions [Kyriakou et al., 2015].

Our analysis demonstrated that DSD phenotypes occur in a clinically significant proportion (∼5%) of DDD participants with undiagnosed learning difficulties, and a range of DSD phenotypes are found in patients with neurodevelopmental delay. Although a high number of children had hypospadias, we found that cryptorchidism in children with undiagnosed neurodevelopmental problems was particularly common. Haire et al. [2015] described a number of teenage boys with severe learning difficulties who developed cryptorchidism later on in life due to their generalized increased muscle tone, raising the possibility that some of these cases of cryptorchidism in our group may have been acquired. It is not clear, however, if all the boys had a detailed early assessment of their external genitalia and whether cryptorchidism may have been overlooked previously due to the complexity of their care. Furthermore, in the current study it was unclear whether the term cryptorchidism included impalpable testes, inguinal testes, or testes that were not fully descended in the scrotal sacs. Given the high incidence of reported cryptorchidism, this will need clarification in future studies, especially since 9 of the 13 (69%) gene mutations identified in this study related to cryptorchidism. It is worth noting that the HPO classification allows the selection of multiple ways of describing the same phenotype, which may necessitate the acquisition of a more enhanced clinical ascertainment in prospective studies.

Of the 13 genes that we found to contain likely pathogenic mutations, 6 (46%) have not previously been reported to be associated with DSD. One of these genes, *ARHGAP31*, is known to be associated with Adams-Oliver syndrome that is characterized by scalp defects and distal limb reduction anomalies. The *ARHGAP31* gene encodes a Rho GTPase activating protein that regulates the RAC1 and CDC42 proteins and is required for normal cell migration [Tcherkezian et al., 2006]. The patient with the mutation has micropenis as well as cryptorchidism. Another newly implicated gene, *CHD2* (chromodomain helicase DNA-binding protein 2), had been described in childhood onset epileptic encephalopathy and myoclonic astatic epilepsy. It has a role in chromatin remodelling, possibly by modification of histone proteins, thus altering access of the transcriptional apparatus to its chromosomal DNA template and therefore altering gene expression [Carvill et al., 2013]. The patient with the *CHD2* mutation has micropenis.

There are multiple ways in which mutations in these genes could result in developmental anomalies such as cryptorchidism and other DSDs. For instance, these genes encode proteins that have been linked to chromatin remodelling, transcriptional activation, protein translation, apoptosis, clathrin-mediated endocytosis, cytoskeletal remodelling, and tube formation. In addition, those genes that encode proteins which directly (*FOXP1*) or indirectly (*CHD2*) regulate gene transcription have the potential to modulate the expression of numerous other genes that may themselves individually play key roles in normal human development.

DSDs cause considerable physical and psychological morbidity in affected individuals and their families. The discovery of new genetic associations will increase our ability to establish molecular diagnosis which will further enhance clinical management and enable accurate genetic counselling for these families. The above cohort represents a small subset of the DDD cohort with DSD features where genetic data were available. Further review of all the genotypic data using more in-depth analysis will allow us to report on additional genetic associations and genetic variations identified in patients with DSD.

Exomic sequencing through projects like DDD increases diagnostic yield, whilst the identification of mutations in developmental genes may improve understanding about the pathogenesis of DSD. Furthermore, increased awareness of these syndromic associations will prompt clinicians to look for associated DSD phenotypes, which will decrease the chances of overlooking DSD in the management of patients with complex conditions.

## Statement of Ethics

The DDD study had previously obtained the relevant national Research Ethics Committee (REC) and Research and Development approvals (10/H0305/83, granted by the Cambridge South REC, and GEN/284/12 granted by the Republic of Ireland REC).

## Disclosure Statement

The authors have no conflicts of interest to declare.

## Figures and Tables

**Table 1:** Genes previously described in association with DSD

Gene	Phenotype	DSD phenotype described previously	DSD phenotype of patient (HPO terms)
*PACS1*	mental retardation	cryptorchidism; Schuurs-Hoeijmaker et al. [2012]	genital hypoplasia
*EP300*	colorectal cancer, somatic, Rubinstein-Taybi syndrome type 2 (RSTS2)	Hypospadias and cryptorchidism; Woods et al. [2014]	cryptorchidism
*KDM6A*	Kabuki syndrome type 2	hypospadias, cryptorchidism, and (more rarely) micropenis, hypoplastic labia; Armstrong et al. [2005]	cryptorchidism
*LRP2*	Donnai-Barrow syndrome	genitourinary abnormality rare – uterine abnormality reported; bicornate uterus (OMIM 222448)	cliteromegaly
*CDT1*	Meier-Gorlin syndrome type 4	cryptorchidism, hypospadias, micropenis, hypoplastic labia; de Munnik et al. [2012]	cryptorchidism
*ANKRD11*	KBG syndrome	cryptorchidism; Tekin et al. [2004]	cryptorchidism
*ATRX*	alpha-thalassemia, myelodysplasia syndrome, somatic alpha-thalassemia, mental retardation syndrome, mental retardation-hypotonic facies syndrome, X-linked	hypospadias, cryptorchidism, underdeveloped scrotum, small penis; Stevenson et al. [2000]	genital hypoplasia

The terms entered in the table with regard to the DSD phenotype of patients are the precise HPO terms that were selected by the recruiting clinical geneticists and subsequently kindly provided to us by the DDD study.

**Table 2:** Genes not previously described in association with DSD

Gene	Phenotype	DSD phenotype of patient
*OCRL*	Dent disease type 2, Lowe syndrome	cryptorchidism
*CHD2*	epileptic encephalopathy, childhood onset	micropenis
*ARHGAP31*	Adams-Oliver syndrome type 1, congenital scalp defects, distal limb reduction anomalies	micropenis, cryptorchidism
*SCN2A*	epileptic encephalopathy, early infantile type 11; seizures, benign familial infantile type 3	cryptorchidism
*RARS2*	pontocerebellar hypoplasia, type 6	cryptorchidism
*FOXP1*	mental retardation with language impairment and autistic features	cryptorchidism
